# The Hospital-Community-Family–Based Telemedicine (HCFT-AF) Program for Integrative Management of Patients With Atrial Fibrillation: Pilot Feasibility Study

**DOI:** 10.2196/22137

**Published:** 2020-10-21

**Authors:** Jiang Jiang, Xiang Gu, Chen-Di Cheng, Hong-Xiao Li, Xiao-Lin Sun, Ruo-Yu Duan, Ye Zhu, Lei Sun, Fu-Kun Chen, Zheng-Yu Bao, Yi Zhang, Jian-Hua Shen

**Affiliations:** 1 Dalian Medical University Dalian China; 2 Medical College of Yangzhou University Yangzhou, Jiangsu China; 3 Department of Cardiology Northern Jiangsu People's Hospital Yangzhou China; 4 Second Affiliated Hospital, Xiang-Ya Medical College of Central South University Changsha China

**Keywords:** atrial fibrillation, integrative management, telemedicine, self-management, feasibility study

## Abstract

**Background:**

The potential effectiveness of integrated management in further improving the prognosis of patients with atrial fibrillation has been demonstrated; however, the best strategy for implementation remains to be discovered.

**Objective:**

The aim of this study was to ascertain the feasibility of implementing integrated atrial fibrillation care via the Hospital-Community-Family–Based Telemedicine (HCFT-AF) program.

**Methods:**

In this single-arm, pre-post design pilot study, a multidisciplinary teamwork, supported by efficient infrastructures, provided patients with integrated atrial fibrillation care following the Atrial fibrillation Better Care (ABC) pathway. Eligible patients were continuously recruited and followed up for at least 4 months. The patients’ drug adherence, and atrial fibrillation–relevant lifestyles and behaviors were assessed at baseline and at 4 months. The acceptability, feasibility, and usability of the HCFT-AF technology devices and engagement with the HCFT-AF program were assessed at 4 months.

**Results:**

A total of 73 patients (mean age, 68.42 years; 52% male) were enrolled in November 2019 with a median follow up of 132 days (IQR 125–138 days). The patients’ drug adherence significantly improved after the 4-month intervention (*P*<.001). The vast majority (94%, 64/68) of indicated patients received anticoagulant therapy at 4 months, and none of them received antiplatelet therapy unless there was an additional indication. The atrial fibrillation–relevant lifestyles and behaviors ameliorated to varying degrees at the end of the study. In general, the majority of patients provided good feedback on the HCFT-AF intervention. More than three-quarters (76%, 54/71) of patients used the software or website more than once a week and accomplished clinic visits as scheduled.

**Conclusions:**

The atrial fibrillation–integrated care model described in this study is associated with improved drug adherence, standardized therapy rate, and lifestyles of patients, which highlights the possibility to better deliver integrated atrial fibrillation management.

**Trial Registration:**

Clinicaltrials.gov NCT04127799; https://clinicaltrials.gov/ct2/show/NCT04127799

## Introduction

Atrial fibrillation is the most prevalent cardiac arrhythmia and has cumulatively been acknowledged as a major health care burden globally [1]. Despite the fact that atrial fibrillation is infrequent among young people (<1% in individuals aged <40 years), its incidence dramatically increases with age, reaching up to 10%-17% in individuals above the age of 80 years [1,2]. The lifetime risk of atrial fibrillation is 21%-23% in women and 17%-26% in men [3,4]. Approximately 33.5 million individuals were suffering from atrial fibrillation in 2010 globally, which is predicted to double by 2050 owing to widespread population aging [5]. Additionally, atrial fibrillation is associated with a 5-fold increase in ischemic stroke risk and accounts for 15%-20% of all strokes [6,7]. Strokes arising from atrial fibrillation are more catastrophic and disabling than those of other etiologies, which has been described as an “atrial fibrillation–correlative stroke tsunami” [8].

Oral anticoagulants therapy can markedly reduce the risk of stroke by 64% and the risk of death by 26% in patients with atrial fibrillation [9]. However, the underuse or improper use of oral anticoagulants is fairly common in real-world clinical practice, even in the new era of nonvitamin K antagonist oral anticoagulants (NOACs), especially in many Asian countries [10-12]. In comparison with Europe (90.1%) and North America (78.3%), the reported usage rate of oral anticoagulants was notably lower in Asia (55.2%) and well below the global average (79.9%) [11]. Furthermore, 17.7% of high-risk patients were not anticoagulated (Europe 8.8%; Asia 42.4%), whereas 76.5% of low-risk patients were inappropriately anticoagulated [11]. In Asia, oral anticoagulant use varies from 21.0% in China (5.8% for NOACs) to 89.7% in Japan [11].

The integrative management of atrial fibrillation patients is deemed to have the potential to improve the oral anticoagulant rate and patient prognosis, which comprises the following core elements: (1) patient-centeredness, (2) multidisciplinary teamwork, (3) utilization of intelligent technology, and (4) application of comprehensive strategies with access to all therapy options [13-15]. Additionally, the European Society of Cardiology developed an app to support the management of patients with atrial fibrillation [16]. To date, several preliminary studies have demonstrated the benefits of integrated atrial fibrillation care in reducing readmission rates [17], cardiovascular and all-cause mortality [17-20], and atrial fibrillation–associated health care expenditures [21]. However, a majority of prior studies have been dominated by nurses [17-19] with latent gaps in the better implementation of guidelines [22]. Additionally, these were mostly single-center studies covering a finite region or supported by a single management strategy such as a structured telephone follow up [23], web platform [16], or app [24].

To our knowledge, no subsistent program has integrated a mobile app, web platform, and intelligent health monitoring devices with cooperation among specialists, general practitioners, patients, and their caregivers to care and empower patients in disease self-management. There are scarce data on the implementation of such an integrated program to manage atrial fibrillation patients, particularly regarding its feasibility and safety. On the basis of our heart failure management program [25,26], we conducted the Hospital-Community-Family–Based Telemedicine (HCFT-AF) program incorporating the core elements mentioned above. The aim of this study was to ascertain the feasibility of the HCFT-AF program for implementation of integrated atrial fibrillation care.

## Methods

### Study Design and Participants

**Figure 1:**
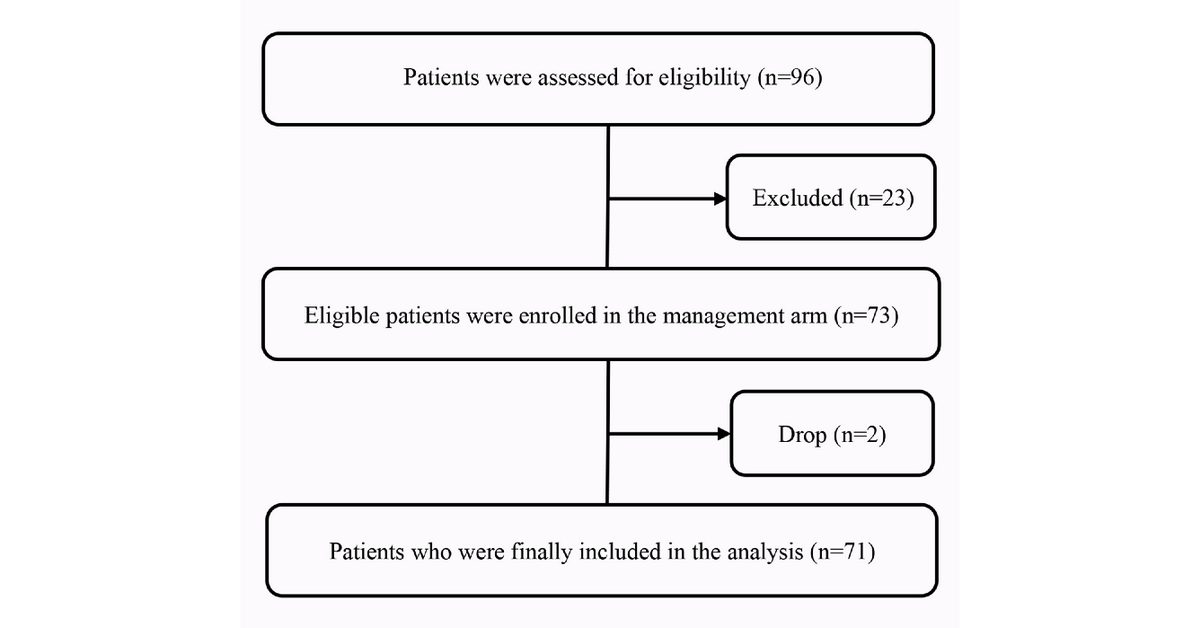
Flow diagram of the pilot study.

This single-arm, pre-post design study was part of the HCFT-AF program (ClinicalTrials.gov NCT04127799), which received ethics approval (2019093) from the Institutional Review Board of Northern Jiangsu People’s Hospital [27]. Eligible patients (Textbox 1) were enrolled consecutively in November 2019 and were followed up for at least 4 months (Figure 1). All patients provided written informed consent before enrolling in the study.

**Inclusion and exclusion criteria.**

**Inclusion criteria**

- Aged≥18 years
- Meeting the diagnostic criteria for atrial fibrillation
- Understand the nature of the study, and agree to sign informed consent and continue follow up

**Exclusion criteria**

- Atrial fibrillation due to a reversible cause (eg, untreated hyperthyroidism, acute myocardial infarction, or acute myocarditis within 1 month)
- No recurrence of atrial fibrillation after surgical treatment
- Combined with other diseases with a life expectancy less than 1 year
- Severe liver and kidney disfunction: serum creatinine>5.0 mg/dL; alanine transaminase exceeds the reference value by more than 3 times (>100 U/L)
- Systolic/diastolic blood pressure≥180/110 mmHg, but can be enrolled after achieving blood pressure control
- Diagnosed or suspected blood system disease (except for mild to moderate anemia), leading to coagulopathy or combined with bleeding tendency
- Pregnant and lactating women
- Unable to use remote monitoring equipment (eg, depression, dementia)
- Participating in other treatment research or remote patient management programs
- Investigators considered that it is inappropriate to participate in the study

### Supportive System in the HCFT-AF Program

#### System Overview

A user-friendly supportive system was developed to better deliver integrated atrial fibrillation management. In general, this system incorporates the following three major components: (1) a multifunctional service platform (Physio-Gate PG 2000, GETEMED AG), (2) a personal health data app (King OPTO-Electronic AG), and (3) a few health monitoring devices.

#### Multifunctional Service Platform

The multifunctional service platform [28] covers patients’ data collection, storage, education, and audio-video portions. In our initial version, patients’ data could only be collected manually, which was time-consuming, labor-intensive, and had a high error rate. In version 2.0, the platform can automatically create a structured medical record via a document exported from the hospital information system. Additionally, data uploaded by participants through the app can be deposited on the platform. Abnormal values are coded with different colors: green denotes reduction, orange denotes moderate risk, red denotes high risk, and no color denotes normal or approximating the normal range. In this way, physicians can master patients’ condition more comprehensively and rapidly. The online library on the platform provides multifaceted knowledge on atrial fibrillation (eg, guideline-based therapy, guidance for self-management). The platform is also equipped with a highly confidential audio-video system to protect patient privacy. In brief, this platform serves as a bridge for information interaction and resource sharing between all stakeholders in this program.

#### Mobile App

A user-friendly mobile app, available on both Android and iOS, was respectively developed for patients and physicians. The data obtained with access to an internet connection can be accessed offline, after the network is disconnected, while subsequent updates require connection to the network. The version for physicians assists them in better managing patients and allows them to observe and track the health status of patients more conveniently. Physicians can view the medical records of patients in their charge. When patients upload data or seek remote consultation, their attending physicians will be reminded.

**Figure 2:**
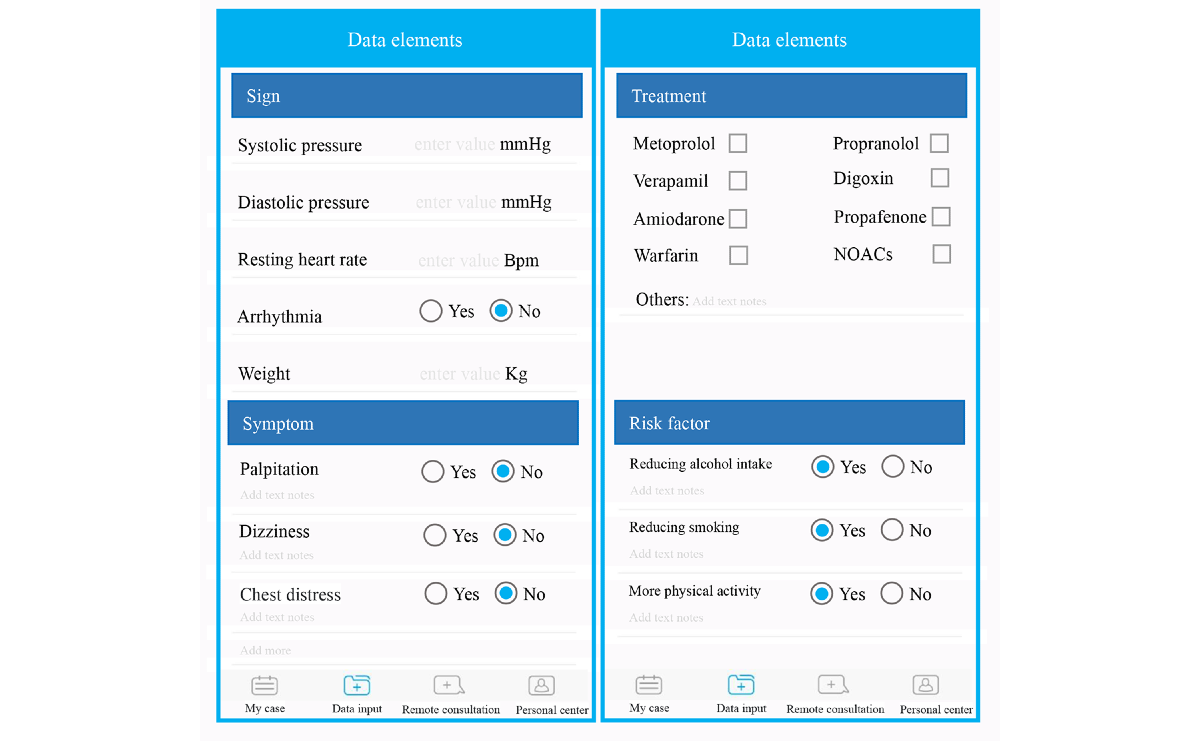
Screenshots of the mobile app for patients.

The patient version has the following major functions: medical record viewing, health-related data uploading, and remote consultation (Figure 2). This app enables patients to view their medical records documented by physicians, which is significant for those taking multiple drugs simultaneously. As an important part of self-management, patients can record and upload health-related data via the app, including daily recording of symptom changes, resting ventricular rate, rhythm, and blood pressure. Their attending physicians will then examine the data and provide corresponding suggestions. In addition, patients can communicate with physicians more conveniently with the remote consultation function. Bleeding caused by warfarin is partly due to unreasonable medication combinations, which can be avoided by timely communication with clinicians [29]. Furthermore, a reminder alert is sent automatically for forthcoming clinic appointments and a structured follow-up schedule is accessible in the app.

#### Intelligent Health Monitoring Devices

In addition to the ACT Plus automatic clotting time tester (Medtronic PLC, Minneapolis, MN, USA), some intelligent health monitoring devices are available in our program: a multicomponent monitor (blood pressure, oxyhemoglobin saturation, and electrocardiograph; TE-4000Y, Beijing Hailiying Medical Technology Ltd, Beijing, China) and a long-term wearable electrocardiograph monitor (BECG1200-A, Thoth Medical Technology Ltd, Suzhou, China) (see Multimedia Appendix 1). These health monitoring devices make remote monitoring, recording, and analysis of patient health status possible.

### Procedures

In general, the intervention included three major elements: education, streamlined guideline-based therapy, and periodic follow up. The educational section mainly had two target audiences. The first was patients and their caregivers, incorporating the detriment and self-management of atrial fibrillation, use of supportive health monitoring devices, and other components. The second was general practitioners in communities, aiming to narrow the gap between physicians and guideline-based therapy.

Researchers introduced the study protocol to the eligible patients and their caregivers. In addition, for integrating the management group, research assistants helped to install the app, and provided instructions on use of the multifunctional service platform, app, and health monitoring devices. A complete medical record was established on the multifunctional service platform before the study officially started. Thereafter, patients were assigned to the community hospital closest to them for further follow up. Structured follow up was arranged once every 2-4 months after discharge, and related examinations were performed every 3-6 months or depending on the condition of patients. During periodic clinical visits, participants were encouraged to record and upload their health-related data via the app and to follow the educational program to improve their disease self-management ability. For patients who were at high risk of severe arrhythmia, some intelligent health monitoring devices were furnished to record and transfer health parameters remotely. Patients and their families could interact with their supervising general practitioners more conveniently via the app if needed.

The general practitioners in community hospitals checked the data submitted by the patients and identified abnormal health data in comparison with previous medical records. In such cases, they would give appropriate treatments or consult specialists at a regional central hospital according to the risk level of the patients. Patients can be transferred to the central hospital through a fast track if necessary. In addition to managing patients, community physicians can also access the latest guideline-based atrial fibrillation therapy through the multifunctional service platform.

Cardiologists of regional central hospitals performed remote ward rounds weekly for patients that were deemed to be complicated or difficult to handle via the dedicated audio-video system by general practitioners, and clinic visit schedules were adjusted according to the condition of the patient when necessary. For patients with complicated conditions, cardiologists would discuss the treatment schedule with neurologists, cardiac surgeons, and other specialists. Furthermore, specialists performed online video seminars regularly for general practitioners and patients, aiming to increase the capacity of general practitioners and compliance of patients to the standardized therapy.

### Outcomes and Instruments

Patients’ drug adherence was assessed via the Pharmacy Quality Alliance adherence measure [30] at baseline and at 4 months individually. Patients’ lifestyles and behaviors associated with the occurrence and progress of atrial fibrillation were collected at baseline and 4 months through interviews with the purpose of evaluating changes in self-management. Additionally, the acceptability, feasibility, and usability of the HCFT-AF intervention for patients were measured via the Perceived Health Web Site Usability Questionnaire [31] at 4 months. This questionnaire consists of three separate portions that evaluate patient satisfaction (eg, “It is easy to find specific information”), ease of use (eg, “I found the HCFT-AF intervention easy to learn”), and usefulness (eg, “Using the HCFT-AF intervention will help me improve my knowledge about health”). All points are rated on a 1 to 7 scale. Responses were averaged for each element and across all points, with higher scores indicating better satisfaction, easier use, higher effectiveness, and greater overall usability of the intervention. The engagement of the HCFT-AF program was roughly estimated by the frequency of patients’ account logins and the completion degree of the follow-up schedule. The patients’ feedback with the intervention was obtained through self-reported questionnaires at the end of the study.

### Statistical Analysis

Demographic traits of patients were collected and are presented as continuous or categorical variables. Continuous variables, verified for normality by the Kolmogorov-Smirnov test, are presented as means (SD) or median (IQR) as appropriate. In addition, the data were compared via a *t* test or Mann-Whitney *U* test, as appropriate. Categorical variables are reported as the absolute number and percentage, which were analyzed by the chi-square test or Fisher exact test. All statistical tests were two-tailed, and *P*<.05 was considered to indicate statistical significance. Statistical analyses were performed on GraphPad Prism version 8.00 for Windows (GraphPad Software, La Jolla, CA, USA).

## Results

### Participant Characteristics

A total of 73 eligible patients (mean age, 68.42 years; 52% male) were enrolled in this feasibility study in November 2019 with a median follow up of 132 days (IQR 125–138). Of the original cohort, 71 patients completed the 4-month follow up. Two patients dropped out after failing to complete regular clinical follow ups. Basic demographic data were collected at baseline (Table 1). Less than half of the qualified patients received anticoagulant therapy. Fifty-three physicians (mean age, 41.72 years; 58% were male) participated in the routine management of patients. Nearly half of the physicians (25/53, 47%) had more than 10 years of work experience. About 30% (16/53) of the physicians were specialists, including cardiologists (20%, 11/53), neurologists (6%, 3/53), and cardiac surgeons (4%, 2/53).

During the study period, specialists from the regional central hospital conducted 34 online lectures and 168 remote ward rounds in total. In addition, general practitioners consulted the experts in the regional central hospital remotely 102 times. One patient with high-grade atrioventricular block detected by intelligent health monitoring devices was transferred to the regional central hospital via a fast track. Furthermore, 5 patients had bleeding gums and 2 had epistaxis; however, the anticoagulation treatment was not interrupted after appropriate treatment was given. No other relevant serious adverse events were reported.

**Table 1:** Baseline characteristics of patients in the study (N=73).

Characteristics		Value
Age (years), mean (SD)		68.42 (10.25)
Male, n (%)		38 (52)
**Medical history, n (%)**		
	Hypertension	37 (51)
	Diabetes	16 (22)
	Congestive heart failure	15 (21)
	Previous stroke/TIA^a^	10 (14)
	Renal dysfunction	6 (8)
	Liver dysfunction	4 (6)
	Peripheral vascular disease	4 (6)
**Type of atrial fibrillation, n (%)**		
	Paroxysmal	35 (48)
	Persistent	28 (38)
	Permanent	10 (14)
**Atrial fibrillation treatment, n (%)**		
	Beta-blocker	34 (47)
	Pharmacologic cardioversion	15 (21)
	Anticoagulant therapy^b^	28 (38)
	Atrial fibrillation ablation	8 (11)
	LAAO^c^	1 (1)
**Anthropometric data, n (%)**		
	BMI (kg/m^2^)	23.15 (5.49)
	Overweight^d^	21 (29)
	Current drinker	36 (49)
	Current smoker	28 (38)
**Daily caregiver, n (%)**		
	Spouse or children	62 (85)
	Relative	8 (11)
	Others	3 (4)
CHA2DS2-VASc^e^ score, mean (SD)		2.89 (1.71)
HAS-BLED^f^ score, mean (SD)		2.32 (1.13)

^a^TIA: transient ischemic attack.

^b^Anticoagulant therapy denotes receiving vitamin K antagonist or nonvitamin K antagonist oral anticoagulant.

^c^LAAO: left atrial appendage occlusion.

^d^Overweight denotes BMI≥25 kg/m^2^.

^e^CHA_2_DS_2_-VASc: congestive heart failure, hypertension, age≥75 (doubled), diabetes mellitus, prior stroke or transient ischemic attack (doubled), vascular disease, age 65-74, female gender.

^f^HAS-BLED: hypertension, abnormal renal/liver function, stroke, bleeding history or predisposition, labile international normalized ratio, elderly, drugs/alcohol concomitantly.

### Predicted Drug Adherence to Long-Term Treatment

Predicted drug adherence of patients significantly improved at 4 months (*P*<.001) (Table 2). More than 90% of the indicated patients received anticoagulant therapy at 4 months, and none of them received antiplatelet therapy unless there was an additional indication.

**Table 2:** Drug adherence, and amelioration in lifestyle and health behaviors.

Variable		Baseline (N=73)	4 months (N=71)	*P* value
Predicted adherence^a^, mean (SD)		6.57 (2.76)	1.45 (1.47)	<.001
**Diet, n (%)**				
	Low-salt, low-fat diet	31 (42)	43 (61)	.04
	More fruits or vegetables intake	18 (25)	54 (76)	<.001
**Healthy lifestyles, n (%)**				
	Moderate physical activity^b^	16 (22)	30 (42)	.009
	Quitting or reducing alcohol intake	37 (51)	52 (73)	.005
	Quitting or reducing smoking	45 (62)	55 (78)	.04
**Self-monitoring, n (%)**				
	Blood pressure	19 (26)	51 (72)	<.001
	Heart rate	8 (11)	37 (52)	<.001
	Rhythm	5 (7)	34 (48)	<.001

^a^Pharmacy Quality Alliance adherence measures were used to predict possible adherence problems at the following three levels: low risk (0), moderate risk (2-7), and high risk (8+); possible range=0-36.

^b^Moderate physical activity=150 minutes/week of moderate-intensity exercise [32].

### Ameliorations in Lifestyles and Healthy Behaviors

The healthy lifestyles and behaviors of patients improved to varying degrees after the 4-month HCFT-AF intervention (Table 2).

### Acceptability, Feasibility, and Usability of the HCFT-AF Intervention

The majority of patients provided good feedback on the HCFT-AF intervention, especially related to its usefulness and satisfaction (Table 3). Cronbach α of the satisfaction, ease of use, usefulness, and overall scale was .89, .85, .83, and .90, respectively. The 31 surveyed physicians (10 specialists and 21 general practitioners) also provided a positive appraisal on the program. All specialists agreed that they were liberated from simple primary repetitive work via the hierarchical management program, and could offer more comprehensive and individual care of patients in comparison with traditional outpatient visits. More than 90% (19/21) of the general practitioners claimed that they had gained substantial professional knowledge and experience about atrial fibrillation from this program. They were also willing and able to provide atrial fibrillation patients with standardized guideline-based therapy.

**Table 3:** Acceptability, feasibility, and usability of patients with the Hospital-Community-Family-Based Telemedicine (HCFT-AF) intervention.

Category^a^	Mean (SD)	Range
Satisfaction	5.21 (1.43)	2.45-7.00
Ease of use	4.76 (1.58)	1.86-7.00
Usefulness	5.45 (1.40)	3.12-7.00
Overall usability of the intervention	5.11 (1.52)	2.68-7.00

^a^Scored on a scale of 1-7; higher scores indicate better satisfaction, easier use, higher effectiveness, and greater overall usability of the intervention.

### Engagement of the HCFT-AF Program

Overall, 76% (54/71) of the patients used the software or website more than once a week and accomplished clinic visits as scheduled. In addition, 70% (50/71) of the patients uploaded health-related data periodically during the 4-month study period. It is worth mentioning that more than half (56%, 40/71) of the patients could utilize the mobile app or website by themselves. Learning education materials, communicating with health care physicians, and viewing their own medication prescriptions were the three most frequently used functions.

## Discussion

### Principal Results

**Figure 3:**
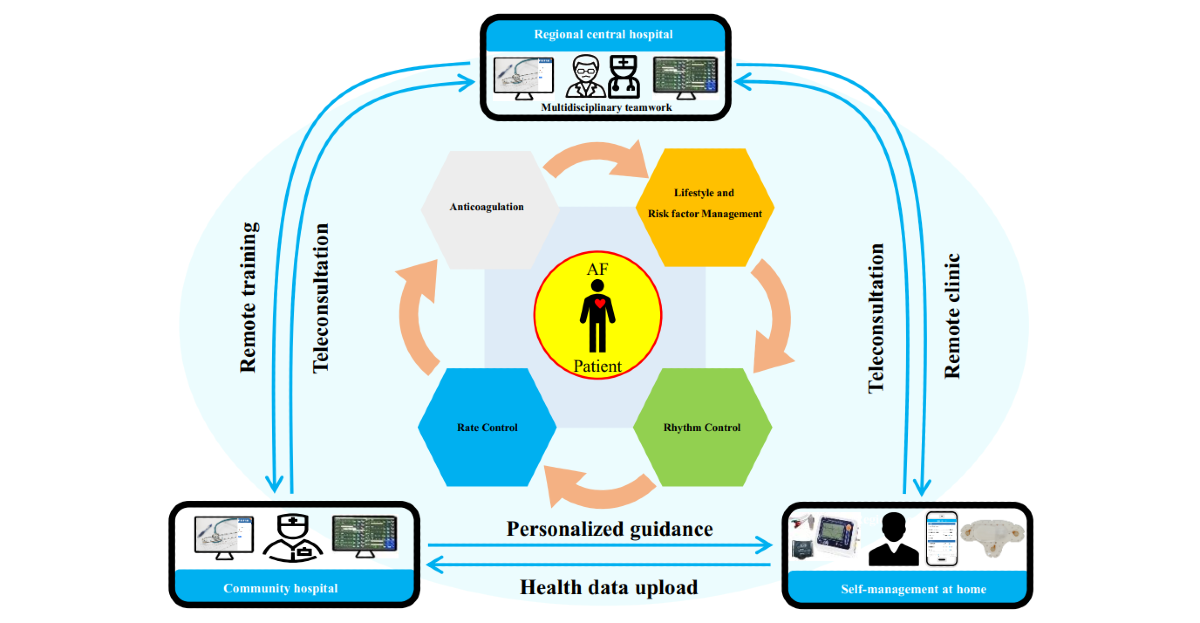
The Hospital-Community-Family–based Telemedicine Program to implement integrated atrial fibrillation care.

The HCFT-AF program integrates streamlined guideline-based therapy, sustaining education (for patients, their caregivers, and general practitioners), and patient self-management with family involvement via a multifunctional telemedicine platform (Figure 3). This prospective single-arm pre-post design pilot study aimed to assess the feasibility and safety of this program. Preliminary results show that patients and physicians have a high degree of satisfaction and participation. More importantly, the standardized therapy rate, drug adherence, and unhealthy lifestyles of patients were all ameliorated to varying degrees after a short-term intervention.

### Comparison With Prior Work

Several large studies have contributed to disclosing the vital epidemiological traits of atrial fibrillation worldwide but also exposed the underuse of guideline-based standardized treatment, especially anticoagulation therapy [1,5,10,11]. Many factors account for this intractable plight. Warfarin’s narrow treatment window, multiple drug and dietary interactions, and frequent laboratory monitoring are inherent defects contributing to the poor adherence to anticoagulant therapy [33]. The development of NOACs has overcome these drawbacks, but these drugs are still not covered by health insurance in most countries, including China, with a daily cost that is 160 to 200 times higher than that of warfarin, thereby hindering their long-term availability. In addition to the low drug-use rate, the discontinuation of medication is another problem that plagues clinicians. The RE-LY study reported that the rates of discontinuation at 1 and 2 years were 14.5% and 21.1% for dabigatran, and 10.2% and 16.6% for warfarin, respectively [34]; real-world data are even less optimistic [35].

Periodical clinical follow up is important to guarantee safe and effective anticoagulant therapy [17,36]. However, traditional outpatient visits mostly depend on the abnormality of patients’ self-perception. Furthermore, the face-to-face outpatient visit mode prevents doctors from fully communicating with patients and providing effective out-of-hospital management. The HCFT-AF program furnishes patients with dual online and offline consistent follow up. Such facilitated medical visits can help to improve patient compliance with long-term therapy, especially for those with limited mobility or living in rural regions. More than three-quarters (76%, 54/71) of patients accomplished medical visits as scheduled during this 4-month study.

Insufficient awareness of the hazard of atrial fibrillation and excessive worry of bleeding caused by anticoagulation therapy hinder nonspecialists from offering indicated patients with standardized therapy [29,37]. Guideline-based integrated atrial fibrillation management encompasses many aspects such as anticoagulant therapy, reversion to sinus rhythm, and controlling the ventricular rate, which are not easy to master for nonspecialists. However, this can also be simply streamlined in line with the Atrial fibrillation Better Care (ABC) pathway: “A” avoiding stroke with anticoagulants; “B” for better symptom amelioration with symptom-directed treatment via rate or rhythm control; and “C” for cardiovascular and comorbidity risk reduction, comprising lifestyle and risk factors management [38]. Promotion of such a strategy offers an opportunity to improve awareness and diagnosis, while empowering clinicians with straightforward decision-making steps that may align with the therapeutic regimen of generalists and specialists. Integrated management following the ABC pathway could reduce more than two-thirds of the all-cause deaths and half of the composite outcomes (eg, death, major bleeding, ischemic stroke, and myocardial infarction) after an average follow up of 6.2 (SD 3.5) years [39]. Moreover, it can reduce health-related costs significantly [40], demonstrating a clear benefit to optimize the management of patients with atrial fibrillation. However, the best strategy for implementation of such a program is still being explored. A majority of prior studies have been dominated by nurses [17-19] with latent gaps in the better implementation of guidelines [22]. Additionally, these were mostly single-center studies covering a finite region or supported by a single management strategy such as structured telephone follow up [23], web platform [16], or app [24]. The HCFT-AF program, supported by efficient infrastructures, may be an effective carrier for the implementation of integrated atrial fibrillation care.

In addition, perceptions of patients and their caregivers about atrial fibrillation will influence their willingness and ability to obey the treatment recommendations [41]. Several studies have supported that better patient understanding is associated with improved health outcomes [42,43]. As holistic management of atrial fibrillation calls for patients to comply with long-term therapy and transform their unhealthy lifestyles, sometimes without a rapid visible benefit, it is important that they better understand their duties in the treatment. Clinicians are responsible for offering evidence-based therapy, whereas the compliance with the therapy is the responsibility of informed and autonomous patients, which is termed “shared accountability” [44]. A prior study demonstrated that a multifaceted educational intervention significantly improved the proportion of oral anticoagulants utilization, and therefore had the potential to ameliorate the outcome of stroke prevention [45]. The HCFT-AF program provides comprehensive education to patients and their caregivers on the risk factors, treatment, and self-management of atrial fibrillation. The app for patients and intelligent health monitoring devices empowers them to participate in their own disease management, thus aiding in improving adherence to long-term therapies.

Accumulating evidence demonstrates that amelioration in obesity [46], physical fitness [47], and hypertension [48], as well as other risk factors such as alcohol consumption, can reduce the atrial fibrillation burden, often to a degree that is not inferior to that of catheter ablation and other invasive methods [38]. Furthermore, interventions for additional risk factors can strengthen the benefits of disease-oriented therapies such as ablation. A recent scientific statement from the American Heart Association emphasized that lifestyle and risk factor management should be integrated as the fourth pillars in addition to the conventional triangle of atrial fibrillation management [38]. Some previous studies reported that the atrial fibrillation burden and severity can be improved through general lifestyle advice [46], weight intervention [46], or moderate exercise [49]. In general, it is currently well recognized that lifestyle and risk factors modification for atrial fibrillation should be managed as chronic diseases requiring multiple repeated interventions to bring forth long-term successful outcomes. Telemedicine contributing to lifestyle improvement has been confirmed in other domains of medicine [50-56]. On the one hand, telemedicine provides patients and their caregivers with sustaining education on the necessity of lifestyle alterations [50,57]. On the other hand, timely feedback from clinicians enhances patients’ motivation to change their unhealthy lifestyles [52]. The HCFT-AF program encourages patients to record and upload their lifestyles through the app, followed by personalized advice from clinicians. Multiple repeated interventions may further motivate patients to change their unhealthy lifestyles.

With the launch of a large-scale atrial fibrillation screening program, the number of atrial fibrillation patients will increase exponentially [58]. Smartphones, computers, and internet-connected devices have become ubiquitous in modern life, which lay a foundation for integrating new methods and novel technologies to provide better care for more patients. A core team (eg, composed of cardiologists) supported by efficient infrastructures serves as an intermediary with other health care specialists, which may achieve optimal management. The overall process comprises a diagnostic assessment, initiation of appropriate guideline-based therapy, sustaining follow up, and education and empowerment of patients and their caregivers in disease self-management. Accordingly, stabilized and sufficiently managed patients can finish subsequent follow up by supported self-management at home or in the community online or offline. If necessary, the patient can be referred to the regional central hospital rapidly. Such integrated management based on telemedicine makes it possible to provide standardized treatment for more patients with limited medical resources.

### Limitations

Some limitations of this study need to be discussed. First, given that this was a feasibility study aiming to lay the foundation for subsequent large-scale research, we adopted a convenience sample size without a formal power calculation. Second, previous similar studies have shown that our outcomes of interest showed no significant changes in the control group [24,45,50]; therefore, we conducted a single-arm study to ascertain the feasibility and safety of the HCFT-AF program. Hence, this study may not have been sufficiently powered to determine the relative benefits of the intervention. In terms of lifestyle management, we simply educated patients on the importance of lifestyle amelioration rather than providing them with a detailed weight loss or exercise plan. On the one hand, the improvement of lifestyle via education has been confirmed in several previous studies [45,50]. On the other hand, this mode will broaden the adaptability of our findings. Certainly, there is an additional benefit that some patients will attain from the protocol-driven lifestyle and risk factor management program [53]. Finally, the impact on the major clinical outcomes (eg, stroke, bleeding, death) will be summarized in our ongoing randomized controlled study, which was not the principle purpose of this study.

### Conclusions

The findings from this pilot study highlight the major role of the HCFT-AF program in improving the standardized therapy rate, drug adherence, and lifestyles of patients with atrial fibrillation, while further enhancing the guideline adherence of clinicians, thus providing a theoretical basis for eventual clinical benefits. A multidisciplinary team, supported by efficient infrastructures, is conducive to narrow the gap between clinical practice and guidelines. The potential effectiveness of integrated management has already been confirmed; however, more studies are needed to ascertain the best strategy to implement the program, which will allow more patients to benefit from optimal evidence-based therapies.

## Appendix

Multimedia Appendix 1 Supportive health monitoring devices.

## Abbreviations

ABC: Atrial fibrillation Better Care
HCFT-AF: Hospital-Community-Family-Based Telemedicine
NOAC: non-vitamin K antagonist oral anticoagulant
