# Supplementary figures and images for: The Hospital-Community-Family–Based Telemedicine (HCFT-AF) Program for Integrative Management of Patients With Atrial Fibrillation: Pilot Feasibility Study
Source: JMIR Mhealth Uhealth. 2020 Oct 21;8(10):e22137. doi: 10.2196/22137 (PMC7641782; doi:10.2196/22137)

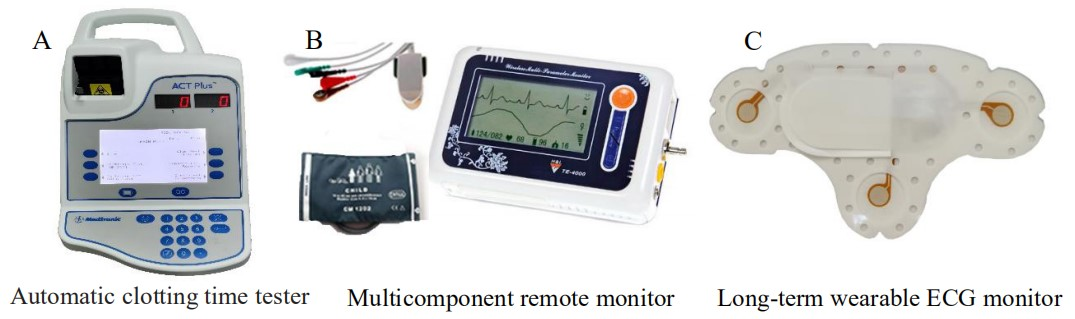

Supplement: Multimedia Appendix 1 [file mhealth_v8i10e22137_app1.png]
